# MIS416 as a siRNA Delivery System with the Ability to Target Antigen-Presenting Cells

**DOI:** 10.1089/nat.2017.0695

**Published:** 2018-08-01

**Authors:** Francesco Mainini, David S. Larsen, Gill A. Webster, Sarah L. Young, Michael R. Eccles

**Affiliations:** ^1^ Department of Pathology, University of Otago, Dunedin, New Zealand.; ^2^ Department of Chemistry, University of Otago, Dunedin, New Zealand.; ^3^ Innate Immunotherapeutics Ltd., Penrose, New Zealand.; ^4^ Maurice Wilkins Center for Molecular Biodiscovery, Auckland, New Zealand.

**Keywords:** dendritic cells, Stat3, siRNA, gene silencing, delivery

## Abstract

MIS416 is a microparticulate formulation derived from *propionibacterium acnes* cell wall skeletons with intrinsic adjuvant activity. Conjugates of MIS416-SS-peptide containing a disulfide linkage facilitate the cytoplasmic delivery and release of peptides in antigen-presenting cells (APCs). We hypothesized that MIS416-siRNA (small interfering RNA) conjugates, containing a disulfide linkage between MIS416 and the siRNA, would allow cytoplasmic release of siRNA in APCs. MIS416-SS-siStat3 conjugates added to cell culture medium of monolayers of DCs in culture flasks successfully targeted *Stat3* mRNA in DCs *in vitro* without transfection, downregulating *Stat3* mRNA and protein levels. These results suggest that MIS416-SS-siRNA conjugates can be used as a novel siRNA delivery system for the knockdown of mRNA levels in APCs.

## Introduction

Recently, small interfering RNAs (siRNAs) have emerged as innovative nucleic acid-based therapies and candidates for the treatment of many diseases [1–4]. However, *in vivo* delivery of siRNA presents many challenges [5]. First, unmodified siRNAs are not stable in serum since they are easily degraded by RNAses, and in addition siRNAs are removed by renal clearance, resulting in a short half-life in blood [6]. Second, siRNAs are impermeable to cells, and a delivery system is required for delivery of siRNAs into the cytoplasm of target cells [7]. Third, siRNAs delivered to cells may become trapped in endosomes, leading to ineffective treatment due to degradation caused by specific DNAses and RNAses [8,9]. To overcome these barriers, siRNA delivery systems need to be designed with the ability to transport and deliver genetic material safely and efficiently. It is also potentially desirable that the delivery vector is able to target specific cells or cell types, with low cytotoxicity.

MIS416 is a bacterial cell wall skeleton derived from *Propionibacterium acnes* comprising multiple nucleotide-binding oligomerization domain-containing 2 (NOD-2) and toll-like receptor 9 (TLR-9) ligands that targets cytosolic receptors expressed by antigen-presenting cells (APCs) [10]. The manufacturing process generates a microparticulate suspension (0.5 × 2.0 μm rods) of minimal cell wall skeleton with bacterial DNA contained within the cage structure. This new delivery platform exploits phagocytic uptake mechanisms to achieve targeted delivery to both myeloid and plasmacytoid DCs and other APCs [10]. Furthermore, the activation of NOD-2 and TLR-9 on APCs results in the upregulation of costimulatory molecules, such as major histocompatibility complex (MHC) I and II, CD86, and CD80 in DCs leading to an effective adaptive immune response in the host [11–13]. The potential use of MIS416 as a therapeutic cancer vaccine adjuvant was recently investigated in a melanoma cancer model [10] and in an epithelial ovarian cancer model [14] in association with CD11b therapy to remove myeloid-derived suppressive cells in the tumor microenvironment. The results showed that MIS416 treatment could delay tumor growth in both murine cancer models, and that MIS416 could synergize with other standard anticancer therapies, such as radiotherapy and with other more novel immunotherapy regimens [14].

We previously developed a conjugation strategy for the coupling of biotinylated peptides and other molecules to MIS416 using a streptavidin bridge [15]. This coupling methodology enabled attachment of fluorophores and peptides to investigate whether the inclusion of a disulfide bond in the linker could facilitate the release of the attached molecular cargos from MIS416. The results showed that inclusion of a disulfide bond in MIS416-SS-peptide conjugates induced more efficient release of peptides in the cytoplasm of DCs, an important consideration for MIS416-mediated delivery of degradation-sensitive cargos such as siRNAs.

Recently, Pradhan *et al.*, reported a nucleic acid therapeutic strategy, involving targeting of dendritic cells (DCs) to influence DC function [16]. In their approach, therapeutic targeting of *IL-10* in DCs was carried out using siRNAs codelivered with adjuvant CpG (a TLR9 ligand) and a pDNA-antigen (the idiotype protein of A20 B cell lymphoma) associated with a PLGA-PEI (poly[lactic-*co*-glycolic acid] and polyethylenimine)-derived microparticle [16]. This approach depends on the inclusion of cationic polymer (PEI) in the PLGA-based formulation to incorporate complexes of siRNAs, CpG, and DNA plasmid by electrostatic interaction, which potentially could be associated with apoptosis of APCs and reduction in the immune response [17,18].

Stat3 is a transcription factor member of the signal transducer and activator of transcription (Stat) protein family. Treatment of immune cells (particularly T cells) with interleukin-6 (IL-6), IL-10, hepatocyte growth factor, epidermal growth factor, as well as other growth factors and cytokines, promotes Stat3 phosphorylation, and consequently Stat3 translocation to the nucleus, where it binds specific DNA sequences and promotes gene expression [19]. Nuclear factor-κB (NF-κB) is a key protein–protein binding partner and transcriptional coregulator of Stat3, and has been linked to the expression of numerous oncogenic and inflammatory genes [20]. Research on nullizygous Stat3^−/−^ immune cells has highlighted the role of Stat3 in inhibiting certain functions of DCs. DCs from Stat3^−/−^ knockout mice exhibited enhanced expression of MHC II, CD80, and CD86 and enhanced antigen crosspresentation [21]. Furthermore, DCs derived from Stat3^−/−^ knockout mice exhibited an improved activation of antigen-specific CD4 T cells *ex vivo* [22]. In contrast, the expression of Stat3 by DCs in the tumor microenvironment inhibited initiation of the adaptive immune response, and led to an immunosuppressive phenotype [23].

In this study, we have investigated the feasibility of conjugating siRNAs to MIS416, using a disulfide linkage (MIS416-SS-siRNA), with the primary objective of delivering functionally active siRNAs to the cytoplasm of APCs to modulate gene expression. We used *Stat3* as a siRNA target [24–27], which showed that MIS416-SS-siRNA conjugates have the potential to deliver siRNAs to APCs, and that MIS-SS-Stat3_siRNA conjugates are able to inhibit *Stat3* mRNA transcription in DCs cultured *in vitro*.

## Materials and Methods

### Preparation of the MIS416-PE conjugate

#### Biotinylation of MIS416

Pellets of MIS416 (10 mg) were washed in sodium bicarbonate (NaHCO_3_) buffer (50 mM, pH = 8.35, 1.5 mL). Sulfo-NHS-biotin (Thermo Scientific) (1.2 mg) was dissolved in NaHCO_3_ buffer (50 mM, pH 8.35, 1 mL) and added to the washed pellet. The mixture was agitated overnight. The supernatant was removed after centrifugation (5,000 *g*, 5 min) and the pellet washed three times with phosphate-buffered saline (PBS) buffer (1.5 mL). The biotinylated microparticle (1 mg) was suspended in PBS (200 μL). An aliquot (20 μL, 4 μg) of streptavidin-PE stock solution (BioLegend) was added and the mixture agitated for 3 h at RT. The resulting pellet was washed in PBS (1.5 mL) twice and centrifuged (5,000 *g*, 5 min). After centrifugation and washing with PBS the pellet was resuspended in PBS (200 μL).

### Preparation of the MIS416-biotin–Streptavidin–biotin-SS-STAT3-FAM (MIS416-SS-Stat3_siRNA) conjugates

The complete conjugation procedure to couple biotinylated molecules to MIS416 has been published previously [15]. Briefly, streptavidin (Invitrogen, 2.5 mg/mL in PBS) was used to conjugate MIS416-biotin particles and biotin-SS-STAT3-FAM (Integrated DNA technology, MW 14591 g/mol). Streptavidin (50 μL, 125 μg, 2.37 nmoles) was added to biotin-SS-STAT3-FAM (20 μL, 69 μg, 4.74 nmoles) in an Eppendorf tube to occupy two of the four biotin-binding sites of Streptavidin. The mixture was agitated for 4 h at 4°C. MIS416-biotin (0.4 mg in 200 μL in NaHCO_3_ buffer, pH 8.3) was added to the mixture and agitated overnight at 4°C. After centrifugation (5,000 *g*, 5 min) and washing with PBS, the pellets were resuspended in PBS (200 μL). The fluorescence of FAM (excitation 488 nm, emission 520 nm) was used to compare multiple preparations of MIS416-SS-STAT3_siRNA. As a negative control, MIS416-SS-control_siRNA (Integrated DNA technology, MW 13389 g/mol) was generated following the procedure just described. The Stat3-specific siRNA sequences were exactly as previously published, 5′-GGGUCUGGCUAGACAAUAUTT-3′ (sense) and 5′-AUAUUGUCUAGCCAGACCCTT-3′ (antisense) [27]. The scrambled control siRNA sequences were 5′-UUCUCCGAACGUGUCACGUTT-3′ (sense) and 5′-ACGUGACACGUUCGGAGAATT-3′ (antisense) [27].

### Internalization of the MIS416-PE conjugate in murine splenocytes

Splenocytes were collected from C57BL/6 mouse spleens. Animal ethics approval was granted by the University of Otago Animal Ethics Committee, protocol numbers ET10/13 and AEC17/14. Cells were washed in PBS (300 *g*, 5 min) and red blood cells were lysed with ammonium chloride buffer (4.15 g NH_4_Cl, 0.5 g KHCO_3_, 0.0186 g EDTA, 500 mL milli-Q water, pH 7.4) for 3 min at 37°C. MIS416-PE (1, 5 or 10 μg) was incubated for 1, 4, or 24 h with 2 × 10^6^ splenocytes at 37°C in 2 mL of the medium. The same protocol was used at 4°C to evaluate the attachment of MIS416-PE to the cell surface (negative control). After the uptake, splenocytes were washed with PBS (300 *g*, 5 min) and stained with CD45R/B220 (clone A3-6B2; BioLegend), CD11c (clone N418; BioLegend), F4/80 (clone BM8; BioLegend), LY6 g (RB6-8C5; BioLegend) and CD3 (clone 17A2; BioLegend) (1 μg/10^6^ cells for each antibody in 100 μL of PBS for 15 min at 4°C). FACS analysis: Cells positive for different markers, including CD45R (B cells), CD11c (DCs), F4/80 (Macrophages), Ly6 g (Neutrophils), CD3 (T cells) were subsequently gated on PE to assess specific internalization of the microparticle formulation (MIS416-PE) (Supplementary Fig. S1; Supplementary Data are available online at www.liebertpub.com/nat).

### Bone marrow-derived cell preparation

Bone marrow was harvested from the femurs and tibias of C57BL/6 mice and red blood cells were lysed with 2 mL of ammonium chloride buffer (4.15 g NH_4_Cl, 0.5 g KHCO_3_, 0.0186 g EDTA, 500 mL milli-Q water, pH 7.4) for 3 min at 37°C. Bone marrow cells were then plated at 3 × 10^6^ cells/well in a six-well plate with 5 mL of complete medium [Iscove's modified Dulbecco's medium (IMDM) (Invitrogen) supplemented with 5% fetal bovine serum (Moregate), and 5 × 10^−5^ M 2-mercaptoethanol (Gibco, Life Technologies)] and cultured for 6 days in the presence of 20 ng mL^−1^ recombinant GM-CSF (ProSpec) in humidified incubators with 5% CO_2_ at 37°C. Every 3 days, half of the cell culture medium of the bone marrow-derived dendritic cell (BMDC) culture was replaced with fresh medium containing the above additives. BMDC was used at day 6 or day 7 when more than 80% of the cells were CD11c^+^.

### Cytokine ELISA assay following BMDC activation using MIS416-SS-siRNA conjugates

After treatment with MIS416-SS-siRNA conjugates, the level of cytokines in the culture supernatants were measured using a standard sandwich ELISA assay. Briefly, 96-well flat-bottomed ELISA plates were coated with 50 μL of corresponding purified antibodies [IL-6, IL-10, TNF-α (BioLegend), IFN-γ (ProSpec) (2 μg mL^−1^; BD Pharmingen)], diluted in coating buffer overnight at 4°C, then washed six times in washing buffer (PBS +0.05% Tween 20). The plates were blocked with 200 μL blocking buffer (PBS +1% BSA) for 2 h at 37°C, then washed six times in washing buffer. Recombinant cytokine standards (concentration of standards from 20 to 20 ng mL^−1^, IL-6, IL10, TNF-α, and IFN-γ) and culture supernatants were diluted in blocking buffer, and 50 μL/well was added to each duplicate well and then incubated for 2 h at 37°C, followed by six washes. Biotinylated antibodies against these interleukins (BD Pharmingen) were diluted in blocking buffer (1 μg mL^−1^), and 100 μL/well was added, then incubated for 1 h at 37°C and then washed six times. Streptavidin–HRP (BioLegend) was diluted 1/3,000 in the blocking buffer, and 100 μL was added to each well, then incubated at room temperature (RT) for 30 min, followed by eight washes. TMB substrate (Life Technologies) was added (100 μL/well), and incubated at RT for color development. Around 1 N H_2_SO_4_ was added (100 μL/well) to stop the reaction. The absorbance was then read by ELISA plate reader (BioTek synergy 2) at 450 nm.

### *In vitro* OT-1 T cell proliferation assay

BMDCs at day 5 were plated (5 × 10^5^ cells/well) in 12-well plates (l mL of complete medium each well) and incubated with MIS416 (0.5 μg) plus SIINFEKL (0.5 μg), MIS416-SS-siStat3 (0.5 μg) plus SIINFEKL (0.5 μg), MIS416-SS-siControl (0.5 μg) plus SIINFEKL (0.5 μg), or untreated. After 24 h of incubation, cells were collected, washed in PBS (300 *g*, 5 min), and plated (5 × 10^4^) in 24-well plates (0.5 mL of complete medium each well) and OT-1 T cells (5 × 10^5^ in 0.5 mL of medium) were added. OT-1 T cells were prepared as described previously [15], and were prestained with the VPD450 proliferative dye (BD Bioscience). Briefly OT-1 T cells were resuspended (1 × 10^6^/mL) in PBS and VPD450 was added to a final concentration of 1 mM. Cells were then incubated at 37°C for 10 min and washed three times in PBS (300 *g*, 5 min) before adding them to DC cultures. After 48 or 72 h of incubation, cells were harvested and stained with infrared near IR-live/dead (Invitrogen, 0.05 μL plus 100 μL of PBS for 15 min at 4°C). After washing in PBS, cells were stained in FACS buffer with CD8 (clone 53–6.7; Cell Lab, Beckman Coulter, Inc.) and CD69 (clone H1.2F3; Cell Lab) (1 μg/10^6^ cells of each antibody in 100 μL of FACS buffer for 15 min at 4°C). Samples were analyzed using a Gallios flow cytometer (Beckman Coulter, Inc.). *In silico* analysis was performed using FlowJo software (version 9; TreeStar, Inc.). The cells were gated for singlets (FSC-H vs. FSC-A), live/dead, and CD8^+^. The CD8^+^ gate was further analyzed using the proliferation software tool in FlowJo version 9 to calculate the percentage of proliferating CD8^+^ OT-1 T cells in each sample.

### Evaluation of *Stat3* mRNA levels

BMDC, 5 × 10^5^, at day 6 with 2 mL of complete IMDM (described in the cell culture section) were plated in a 12-well plate. MIS416-SS-STAT3_siRNA or MIS416-SS-BIM_siRNA (3 μg), MIS416 (3 μg), and MIS416-SS-control_siRNA (3 μg) were added in separate wells, whereas one well with untreated cells was used as a control. MIS416 conjugates were incubated for 48 or 72 h. RNA was extracted 48 or 72 h after siRNA treatment using the Ambion RNA Extraction Kit (Life Technologies) according to the manufacturer's instructions. RNA was quantified using a NanoDrop ND 1000 spectrophotometer. cDNA preparations were performed with the Superscript Vilo cDNA Synthesis Kit (Invitrogen) according to the manufacturer's instructions. Analysis by quantitative real-time PCR (Q-RT-PCR) of cDNA samples, was performed on a LightCycler 480 (Roche) using LightCycler 480 SYBR Green Master Mix assays (Roche) according to the manufacturer's instructions. The program used for the LightCycler480 was: start (1 cycle, 2 min 95°C), amplification (40 cycles, 30 s 95°C, 1 min 60°C), dissociation (95°C continuous). *Bim, Stat3*, *B2 m, ß-actin, Ywhaz, Rpl32*, *Minor*, and *IL10r1* cDNA levels (Supplementary Figs. S2 and S3) were quantified using *Gapdh* as the reference gene. Primer sequences are listed in Supplementary Table S1. The relative quantification was carried out using BioGazelle qBase+software.

### Evaluation of *Stat3* protein levels

BMDC, 2 × 10^6^, at day 6 with 5 mL of complete IMDM (described in the BMDC preparation section) were plated in a 12-well plate. MIS416-SS-STAT3_siRNA or MIS416-SS-BIM_siRNA (3 μg), MIS416 (3 μg), and MIS416-SS-control_siRNA (3 μg) were added in separate wells, whereas one well with untreated cells was used as a control. MIS416 conjugates were incubated for 48 or 72 h. Proteins were extracted using RIPA buffer [50 mM Tris-HCl, pH 7.4, 150 mM sodium chloride, 1 mM ethylenediaminetetraacetic acid (EDTA), 1% NP-40, 1% sodium deoxycholic acid, 0.1% sodium dodecyl sulfate (SDS)] with the addition of complete protease inhibitor (cOmplete mini™; Roche), phenylmethylsulfonyl fluoride 1 mM and Na Orthovanadate 1 mM. Western blots were performed on Bolt 4–12% Bis-tris Plus acrylamide gels using Bolt Mes SDS running buffer. Electrophoresis was carried out for 40 min at 165 V. Proteins were transferred using an iBlot^®^ Gel Transfer Device according to the manufacturer's instructions. Nitrocellulose membranes were blocked with 5% skim milk in PBST (PBS, 0.1% TRITON) for 1 h. Anti-beta Actin antibody diluted 1:10,000 (ab8227; ABCAM) and Stat3 (79D7) rabbit mAb diluted 1:2,000 (Cell Signaling Technology) were used to evaluate protein loading and to quantify Stat3 expression levels, respectively. Primary antibodies were detected with anti-rabbit HRP diluted 1:20,000 (A0545; Sigma) and ECL analysis was performed using Super Signal West Pico reagents according to the manufacturer's instructions. Kodak BioMax XAR Films were developed using a Protec ECOMAX X-Ray Film Processor. Developed films from western blots were analyzed using ImageJ software to quantify band density. The ratio between Stat3 and β-Actin band density was used to define a relative density. A value of 1 has been arbitrarily attributed to cells treated with MIS416-SS-control_siRNA and has been used to compare relative density values of cells treated with MIS416 conjugates.

### Statistical analyses

All statistical analyses were performed using GraphPad Prism software version 6. Two-way ANOVA analysis with multiple comparison (Bonferroni) was used to analyze grouped column graphs. Error bars represent the standard error of the mean (SEM).

## Results

### MIS416-PE conjugate is internalized by murine APC populations derived from splenocytes

MIS416 has been described as a microparticulate vector able to deliver attached cargo to DCs and other APCs [15]. To assess the targeting specificity of MIS416 in APCs, the uptake efficiency of MIS416 in various cell populations of the spleen was analyzed using flow cytometry. Murine splenocytes were pulsed with MIS416–Phycoerythrin (PE) conjugate (a fluorescent version of MIS416) for 1, 4, or 24 h at various concentrations, after which they were analyzed by flow cytometry. Different cell populations (macrophages, DCs, T cells, neutrophils, and B cells) were identified with specific antibodies (F480, CD11c, CD3, LY6, and B220), respectively. A 4°C control was used to evaluate nonspecific binding of MIS416-PE to the cell surface.

**FIG. 1.**
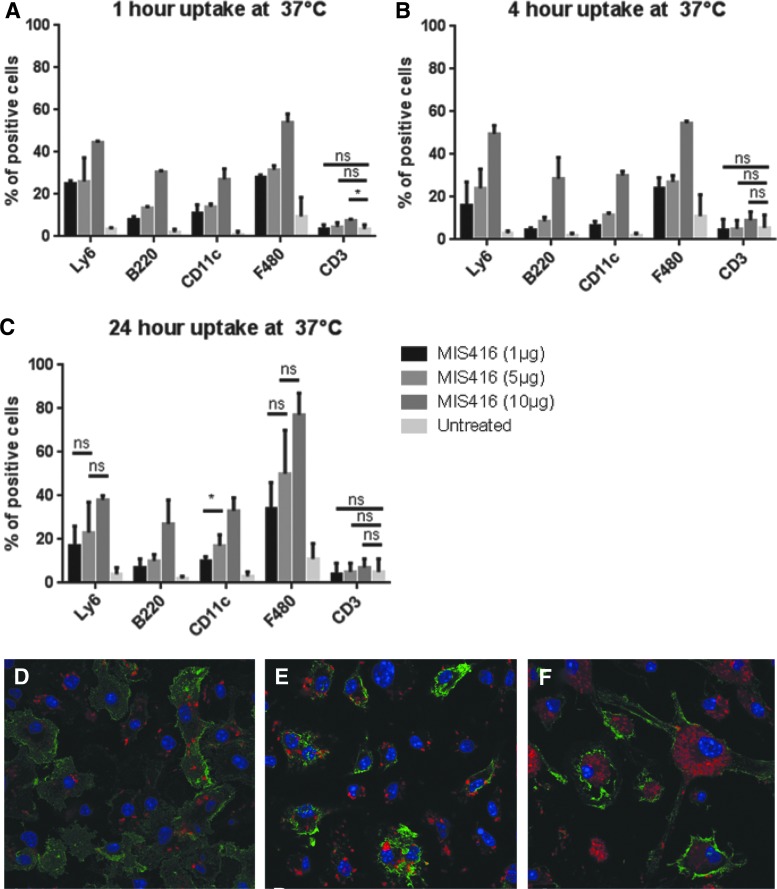
Internalization of MIS416-PE (MIS416-biotin-streptavidin-PE) by splenocytes, and BMDCs. **(A–C)** Graphs showing internalization of MIS416-PE after 1, 4, or 24 h, respectively, at 37°C. Splenocytes were pulsed with MIS416-PE (1, 5, or 10 μg) for 1, 4, or 24 h. After incubation, cells were washed in PBS, and then immunocytochemically stained with five different antibodies in PBS (LY6, B220, CD11c, F480, CD3) to identify five different cell populations (neutrophils, B cells, DCs, macrophages, and T cells), respectively. The gating strategies are explained in Supplementary Fig. S1. Error bars represent standard error of the mean (SEM). ns, not significant; *P < 0.05; **P < 0.005; ***P < 0.0005. The experiment was repeated three times. **(D–F)** Images showing BMDCs at 1, 4, and 24 h, respectively, after internalization of MIS416-PE. BMDCs (2 × 10^5^on coverslips in 250 μL of cell culture media) were treated with MIS416-PE (3 μg) for 1, 4, and 24 h. After incubation, cells were washed in PBS, and immunocytochemically stained with CD11c to identify DCs by incubating 1 μg/10^6^ cells in 100 μL of PBS for 30 min at 4°C. CD11c is shown in *green*, MIS416-PE in *red*, and DAPI (to stain nuclei) in *blue*. The images were taken using a Zeiss LSM 710 confocal microscope. BMDC, bone marrow-derived dendritic cell; PE, phycoerythrin.

Macrophages, DCs, neutrophils and B cells, but not T cells, internalized MIS416-PE (Fig. 1), with approximately 80% of the macrophages and approximately 40% of neutrophils, B cells, and DCs positive for MIS416-PE after 24 h of treatment at 37°C. No major differences in internalization were observed from 1 to 4 h following treatment, indicating that most of the microparticle was internalized within the first hour *in vitro* at 37°C. Robust MIS416-PE fluorescence was observed after 24 h of treatment with MIS416-PE. Internalization of MIS416-PE by macrophages increased by an additional ∼20% from 4 to 24 h of treatment, which was dependent on MIS416-PE concentration. Confocal microscopy using BMDCs additionally showed that MIS416-PE was readily internalized by BMDC after 1 h (Fig. 1D–F) and further MIS416-PE accumulation in the cytoplasm was observed at later time points. Increasing concentrations of MIS416-PE (1, 5, 10 μg) directly correlated with increasing degrees of internalization in splenocytes. Experiments carried out at 4°C, assuming either very little or no internalization at this temperature, showed that approximately 10% of the signal at 37°C was probably due to MIS416-PE binding to the surface of target cells as opposed to being internalized. Macrophages showed a higher signal at 4°C at all time points tested, suggesting they were more prone to cell surface binding effects at 4°C than other cell types.

### MIS416-SS-Stat3_siRNA downregulates *Stat3* mRNA and protein levels in BMDCs

**FIG. 2.**
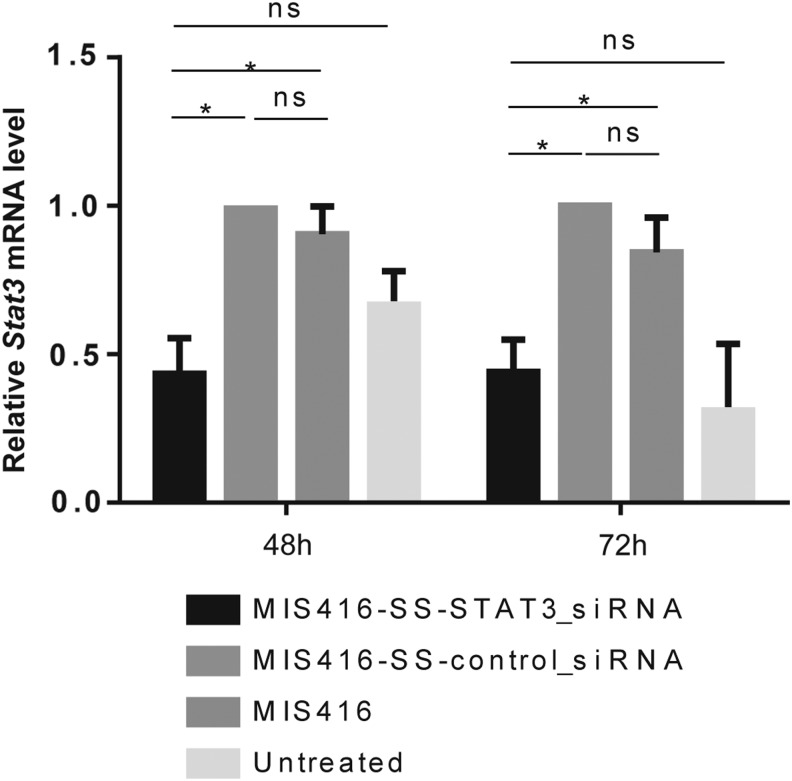
Q-RT-PCR quantification of *Stat3* mRNA levels. DCs were treated for 48 or 72 h with MIS416, MIS416-SS-Control_siRNA, and MIS416-SS-Stat3_siRNA using previously published sequences for *Stat3*-targeting siRNAs [27]. Analysis by Q-RT-PCR was performed on cDNA generated from total RNA extracted from treated cells. Downregulation of *Stat3* mRNA levels occurs at 48 and 72 h in all samples treated with MIS416-SS-Stat3_siRNA compared with controls. The relative level of expression of *Stat3* was set to 1 for MIS416-SS-Control_siRNA samples. The relative quantification was carried out using BioGazelle qBase+software. Error bars represent SEM. Results designated with ns were not significant. Results designated with * were significant (*P* < 0.05). This experiment was repeated three times.

We evaluated downregulation of *Stat3* mRNA and protein *in vitro* following 48 or 72 h of treatment of murine BMDCs with MIS416-SS-Stat3_siRNA conjugates, prepared according to our previously described method [15], compared with treatment with MIS416 alone or conjugated to control siRNA (MIS416-SS-control_siRNA). Analysis by Q-RT-PCR revealed significant reduction of the *Stat3* mRNA levels to approximately 50% of the level of control treated cells, between DCs treated *in vitro* with MIS416-SS-Stat3_siRNA versus treatment of DCs with MIS416-SS-control_siRNA at 48 (*P* < 0.05) and 72 h (*P* < 0.05) (Fig. 2).

**FIG. 3.**
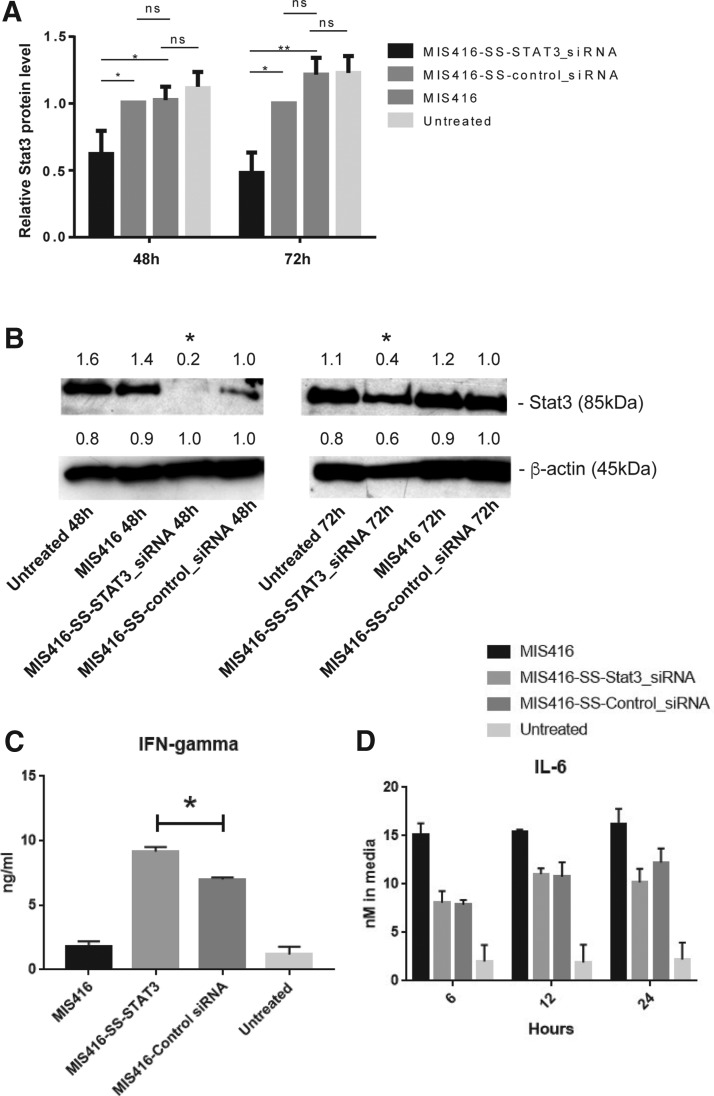
Western blot quantification of Stat3 protein levels. **(A)** Graphs showing the relative Stat3 protein levels following treatment of DCs for 48 or 72 h with MIS416, MIS416-SS-Control_siRNA, or MIS416-SS-Stat3_siRNA. Relative Stat3 protein levels were quantified using ImageJ software. **(B)** Shows an example of western blot of data used to quantify Stat3 protein levels in **(A)**. The numbers above the gel lanes represent the relative protein level, which was determined from the band intensity using ImageJ software, and normalized relative to the MIS416-SS-control_siRNA-treated samples. **(C)** Graph of IFN-γ cytokine levels following treatment of BMDCs for 24 h with MIS416, MIS416-SS-Stat3_siRNA, MIS416-SS-Control_siRNA, or no treatment. **(D)** Graph of ELISA assays of IL-6 cytokine levels in BMDCs at 6, 12, and 24 h following treatment with MIS416, MIS416-SS-Stat3_siRNA, MIS416-SS-control_siRNA, or no treatment. In all graphs, the error bars represent SEM, and the results designated with ns were not significant, whereas results designated with * were significant (*P* < 0.05), or ** (*P* < 0.005). These experiments were repeated three times.

Western blot studies were performed to evaluate Stat3 protein levels after treatment of the murine BMDCs with MIS416, MIS416-SS-Stat3_siRNA, or MIS416-SS-control_siRNA, and relative Stat3 expression levels were quantified using β-actin for gel loading normalization. DCs treated with MIS416-SS-Stat3_siRNA expressed Stat3 protein at 48% of the level in MIS416-SS-control_siRNA-treated DCs at 48 (*P* < 0.05), and at 60% of the level of Stat3 protein in MIS416-SS-control_siRNA-treated DCs at 72 h (*P* < 0.05) (Fig. 3A, B).

### Immunomodulatory effects of MIS416-SS-siRNA conjugate treatment of BMDCs

We next used ELISA assays to evaluate changes in the expression of cytokines, such as IFN-γ, IL-6, IL-10, and TNFα in BMDCs following treatment with MIS416-SS-siRNA conjugates. Increased levels of IFN-γ at 24 h were observed following treatment with MIS416-SS-Stat3_siRNA relative to MIS416-SS-control_siRNA conjugates (Fig. 3C). IL-6 and IL-10 levels were investigated at 6, 12, and 24 h following treatment of the BMDCs with MIS416-SS-Stat3_siRNA, MIS416-SS-control_siRNA, or MIS416 alone. The levels of IL-6 showed relatively little difference between the MIS416-SS-Stat3_siRNA and MIS416-SS-control_siRNA conjugate treatments, but IL-10 levels showed a significant increase at 6, 12, and 24 h with the MIS416-SS-Stat3_siRNA treatment relative to MIS416-SS-control_siRNA treatment (Fig. 3D and Supplementary Fig. S4A). There were no significant differences observed in T cell proliferation between MIS416-SS-Stat3_siRNA or MIS416-SS-control_siRNA conjugates, as determined in OT-1 T cell proliferation assays in the presence of SIINFEKL stimulation of T cells (Supplementary Fig. S4B). Lastly, a slight decrease in TNFα levels at 6 and 12 h was observed following treatment with MIS416-SS-Stat3_siRNA relative to MIS416-SS-control_siRNA conjugates (Supplementary Fig. S4C).

## Discussion

In this study, we report the development of an MIS416-SS-siRNA conjugate to deliver functional siRNAs with the aim of achieving RNAi-mediated silencing and downregulation of gene expression in DCs. The MIS416-SS-siRNA microparticle conjugates successfully delivered functionally active siRNAs targeting *Stat3* expression, resulting in downregulation of *Stat3* mRNA and protein levels in DCs. Potential future applications of this technology could include MIS416-SS-siRNA-induced RNAi targeted to DCs to modulate DC function [16,29–31].

We chose *Stat3* as the siRNA target in the present study for the following reasons; (1) *Stat3* was known to be involved in modulating immune responses [21–23]; (2) *Stat3* was known to be expressed in the subpopulations of cells targeted by the MIS416 (ie, APCs) [10]; (3) in prior analysis of *Stat3* mRNA and protein expression characteristics we identified that efficient siRNA-mediated knockdown of *Stat3* in DCs was achievable; and (4) as determined before starting the siRNA experiments, treatment of APCs with MIS416 alone did not result in downregulation of *Stat3* mRNA levels. With respect to the latter, we investigated the effect of MIS416 on the mRNA levels of four candidate genes (*Minor*, *Bim*, *IL10R1*, and *Stat3*) as possible targets for siRNAs in DCs [32,33]. The mRNA levels of the immune regulator *IL-10R1* and the apoptosis regulators *Bim* and *Minor* were downregulated when DCs were treated with MIS416 (Supplementary Fig. S2), which is consistent with activation of a nonimmunosuppressive phenotype. However, the reduced mRNA levels caused by MIS416 would mask the effects of siRNAs on the mRNA level.

In contrast to the inhibitory effects of MIS416 on *Bim* and *Minor* mRNA levels, *Stat3* mRNA levels were upregulated (Fig. 2), and Stat3 protein levels remained relatively unchanged after treatment with MIS416 (Fig. 3). Treatment of APCs with MIS416 has previously been found to induce IL-6 and IL-10 (Fig. 3, and see ref 23), which in turn stimulates increased *Stat3* mRNA levels and Stat3 protein phosphorylation [34]. Therefore, while we have shown that MIS416-SS-Stat3_siRNA conjugates inhibit *Stat3* mRNA expression, the stimulatory effects of MIS416 on *Stat3* expression and phosphorylation could potentially counteract this.

Nevertheless, we observed a significant increase in IFN-γ levels, which is thought to result from the exposure in the cytoplasm of the cells to the *Stat3* and *Control* double-stranded siRNA sequences present in the conjugates. IL-10 levels also increased, but there was no change in IL-6 levels in BMDCs following the MIS-SS-Stat3_siRNA treatment. The observation of relatively low levels of TNFα cytokine activation, and the observation that there was relatively little difference in the OT-1 T cell proliferation assays between treatments, suggest that cell death was unlikely to be a major cause of the altered cytokine levels. Our findings that IL-10 levels were significantly increased by the MIS416-SS-Stat3_siRNA conjugate, and IL-6 levels were relatively unchanged compared with the MIS416-SS-control_siRNA, were very similar to the results observed in previous studies, where IL-10, but not IL-6 levels, was significantly increased in DCs in mice as a result of a *Stat3* knockout mutation [35]. Our results are therefore consistent with the suggestion that the *Stat3* mRNA and protein levels in BMDCs were downregulated as a result of the silencing effect of the MIS416-SS-Stat3_siRNA conjugate.

In the present study, there were no differences in T cell proliferation between T cells treated with MIS416-SS-Stat3_siRNA plus SIINFEKL, compared with treatment with MIS416 (or MIS416-SS-control_siRNA) plus SIINFEKL in OT-1 T cell assays. We have previously investigated the immunomodulatory activity of MIS416 conjugates, which contained disulfide-linked SIINFEKL *in vitro* and *in vivo* [15]. In our previous *in vivo* investigations, we observed less immunostimulatory activity with a SIINFEKL conjugate linked to MIS416 with disulfide bond, than with a conjugate containing an irreversible, noncleavable bond [15]. These results suggested that the disulfide bond linking MIS416 and siRNA was probably not stable in extracellular tissues *in vivo*. Conjugates containing an alternative cleavable linkage between MIS416 and cargo, which are more stable than a disulfide bond *in vivo*, should therefore be sought for in *in vivo* applications. We have not investigated the stability of siRNAs conjugated to MIS416 (as MIS416-SS-Stat3_siRNA conjugates) in serum, or *in vivo*, due to the expected instability of the disulfide bond. In this regard, chemical modifications of the siRNAs, such as phosphorothioate, or boranophosphate, together with alternative cleavable linkages in the conjugate, would be helpful to improve the stability of MIS416-siRNA conjugates for *in vivo* applications.

Pradhan *et al.* targeted *IL-10* in DCs using siRNAs codelivered with adjuvant CpG (a TLR9 ligand) plus pDNA antigen, encoding the idiotype protein of A20 B cell lymphoma, associated with PLGA-PEI-derived microparticles [16]. These microparticles were composed of the anti-*IL10* siRNAs, which together with adjuvant and pDNA antigen, inhibited the production of IL-10 in DCs, and improved the survival of treated mice in an A20 B-lymphoma xenograft model. The delay in tumor growth in their experiments was found to result from the inclusion of the anti-*IL10* siRNA in the conjugate. In another recent study, Zhang *et al.*, linked adjuvant CpG to a *Stat3*-targeting decoy oligodeoxynucleotide and achieved significant regression of acute myeloid leukemia in a murine model [36].

## Conclusions

We describe, in this study, MIS416 conjugates with the aim of delivering functional siRNAs to APCs. MIS416-SS-Stat3_siRNA conjugates, which contained a Stat3 siRNA, inhibited *Stat3* mRNA, and protein expression in murine BMDCs treated *in vitro* without using transfection. These conjugates could therefore effectively deliver functional siRNAs to DCs *in vitro*, resulting in RNAi-mediated gene knockdown.

## Supplementary Material

Supplemental data

## References

[1] Palanca-Wessels MC, Booth GC, Convertine AJ, Lundy BB, Berguig GY, Press MF, Stayton PS and Press OW (2016). Antibody targeting facilitates effective intratumoral siRNA nanoparticle delivery to HER2-overexpressing cancer cells. Oncotarget 7:9561–9575 10.18632/oncotarget.7076 PMC4891060 26840082

[2] Gu S, Hu Z, Ngamcherdtrakul W, Castro DJ, Morry J, Reda MM, Gray JW and Yantasee W (2016). Therapeutic siRNA for drug-resistant HER2-positive breast cancer. Oncotarget 7:14727–14741 26894975 10.18632/oncotarget.7409 PMC4924747

[3] Mao M, Chen J, Li X and Wu Z (2015). siRNA-TMEM98 inhibits the invasion and migration of lung cancer cells. Int J Clin Exp Pathol 8:15661–15669 26884835 PMC4730048

[4] Maduri S (2015). Applicability of RNA interference in cancer therapy: current status. Indian J Cancer 52:11–21 26837960 10.4103/0019-509X.175598

[5] Draghici B and Ilies MA (2015). Synthetic nucleic acid delivery systems: present and perspectives. J Med Chem 58:4091–4130 25658858 10.1021/jm500330k

[6] Brenner MK, Gottschalk S, Leen AM and Vera JF (2013). Is cancer gene therapy an empty suit? Lancet Oncol 14:e447–e456 24079872 10.1016/S1470-2045(13)70173-6 PMC3916772

[7] Wittrup A and Lieberman J (2015). Knocking down disease: a progress report on siRNA therapeutics. Nat Rev Genet 16:543–552 26281785 10.1038/nrg3978 PMC4756474

[8] Dominska M and Dykxhoorn DM (2010). Breaking down the barriers: siRNA delivery and endosome escape. J Cell Sci 123(Pt 8):1183–1189 20356929 10.1242/jcs.066399

[9] Ma D (2014). Enhancing endosomal escape for nanoparticle mediated siRNA delivery. Nanoscale 6:6415–6425 24837409 10.1039/c4nr00018h

[10] Girvan RC, Knight DA, O'loughlin CJ, Hayman CM, Hermans IF and Webster GA (2011). MIS416, a non-toxic microparticle adjuvant derived from Propionibacterium acnes comprising immunostimulatory muramyl dipeptide and bacterial DNA promotes cross-priming and Th1 immunity. Vaccine 29:545–557 21034827 10.1016/j.vaccine.2010.10.040

[11] Boćko D, Kosmaczewska A, Ciszak L, Teodorowska R and Frydecka I (2002). CD28 costimulatory molecule–expression, structure and function. Arch Immunol Ther Exp (Warsz) 50:169–177 12098932

[12] Ferrand J and Ferrero RL (2013). Recognition of extracellular bacteria by NLRs and its role in the development of adaptive immunity. Front Immunol 4:344 24155747 10.3389/fimmu.2013.00344 PMC3801148

[13] He X, Jia H, Jing Z and Liu D (2013). Recognition of pathogen-associated nucleic acids by endosomal nucleic acid-sensing toll-like receptors. Acta Biochim Biophys Sin Shanghai 45:241–258 23369718 10.1093/abbs/gms122 PMC7109797

[14] Khan ANH, Kolomeyevskaya N, Singel KL, Grimm MJ, Moysich KB, Daudi S, Grzankowski KS, Lele S, Ylagan L, et al. (2015). Targeting myeloid cells in the tumor microenvironment enhances vaccine efficacy in murine epithelial ovarian cancer. Oncotarget 6:11310–11326 25888637 10.18632/oncotarget.3597 PMC4484458

[15] Mainini F, Larsen DS, Webster GA, Young SL and Eccles MR (2015). Bridging small molecules to modified bacterial microparticles using a disulphide linkage: MIS416 as a cargo delivery system. PLoS One 10:e0145403 26695183 10.1371/journal.pone.0145403 PMC4687933

[16] Pradhan P, Qin H, Leleux D, Gwak IS, Kwak LW, Roy K, Roy K (2014). The effect of combined IL10 siRNA and CpG ODN as pathogen-mimicking microparticles on Th1/Th2 cytokine balance in dendritic cells and protective immunity against B cell lymphoma. Biomaterials 35:5491–5504 24720881 10.1016/j.biomaterials.2014.03.039 PMC4747034

[17] Khansarizadeh M, Mokhtarzadeh A, Rashedinia M, Taghdisi SM, Lari P, Abnous KH and Ramezani M (2016). Identification of possible cytotoxicity mechanism of polyethylenimine by proteomics analysis. Hum Exp Toxicol 35:377–387 26134983 10.1177/0960327115591371

[18] Morimoto K, Nishikawa M, Kawakami S, Taghdisi SM, Lari P, Abnous KH, Ramezani M, Hashida M (2003). Molecular weight-dependent gene transfection activity of unmodified and galactosylated polyethyleneimine on hepatoma cells and mouse liver. Mol Ther 7:254–261 12597914 10.1016/s1525-0016(02)00053-9

[19] Song L, Turkson J, Karras JG, Jove R and Haura EB (2003). Activation of Stat3 by receptor tyrosine kinases and cytokines regulates survival in human non-small cell carcinoma cells. Oncogene 22:4150–4165 12833138 10.1038/sj.onc.1206479

[20] Yu H, Kortylewski M and Pardoll D (2007). Crosstalk between cancer and immune cells: role of STAT3 in the tumour microenvironment. Nat Rev Immunol 7:41–51 17186030 10.1038/nri1995

[21] Kortylewski M, Kujawski M, Wang T, Wei S, Zhang S, Pilon-Thomas S, Niu G, Kay H, Mulé J, et al. (2005). Inhibiting Stat3 signaling in the hematopoietic system elicits multicomponent antitumor immunity. Nat Med 11:1314–1321 16288283 10.1038/nm1325

[22] Wang T, Niu G, Kortylewski M, Burdelya L, Shain K, Zhang S, Bhattacharya R, Gabrilovich D, Heller R, et al. (2004). Regulation of the innate and adaptive immune responses by Stat-3 signaling in tumor cells. Nat Med 10:48–54 14702634 10.1038/nm976

[23] Rébé C, Végran F, Berger H and Ghiringhelli F (2013). STAT3 activation: a key factor in tumor immunoescape. JAK-STAT 2:e23010 24058791 10.4161/jkst.23010 PMC3670267

[24] Sanseverino I, Purificato C, Varano B, Conti L, Gessani S and Gauzzi MC (2014). STAT3-silenced human dendritic cells have an enhanced ability to prime IFNγ production by both αβ and γδ T lymphocytes. Immunobiology 219:503–511 24674241 10.1016/j.imbio.2014.02.012

[25] Melillo JA, Song L, Bhagat G, Blazquez AB, Plumlee CR, Lee C, Berin C, Reizis B and Schindler C (2010). Dendritic cell (DC)-specific targeting reveals Stat3 as a negative regulator of DC function. J Immunol 184:2638–2645 20124100 10.4049/jimmunol.0902960 PMC3099405

[26] Nefedova Y, Huang M, Kusmartsev S, Bhattacharya R, Cheng P, Salup R, Jove R and Gabrilovich D (2004). Hyperactivation of STAT3 is involved in abnormal differentiation of dendritic cells in cancer. J Immunol 172:464–474 14688356 10.4049/jimmunol.172.1.464

[27] Alshamsan A, Haddadi A, Hamdy S, Samuel J, El-Kadi AO, Uludağ H and Lavasanifar A (2010). STAT3 silencing in dendritic cells by siRNA polyplexes encapsulated in PLGA nanoparticles for the modulation of anticancer immune response. Mol Pharm 7:1643–1654 20804176 10.1021/mp100067u

[28] Chen Q, Wang H, Liu Y, Song Y, Lai L, Han Q, Cao X and Wang Q (2012). Inducible microRNA-223 down-regulation promotes TLR-triggered IL-6 and IL-1β production in macrophages by targeting STAT3. PLoS One 7:e42971 22937006 10.1371/journal.pone.0042971 PMC3427313

[29] Jadidi-Niaragh F, Atyabi F, Rastegari A, Kheshtchin N, Arab S, Hassannia H, Ajami M, Mirsanei Z, Habibi S, et al. (2017). CD73 specific siRNA loaded chitosan lactate nanoparticles potentiate the antitumor effect of a dendritic cell vaccine in 4T1 breast cancer bearing mice. J Control Release 246:46–59 27993599 10.1016/j.jconrel.2016.12.012

[30] Dong Z, Chen Y, Peng Y, Wang F, Yang Z, Huang G, Chen Y, Yuan Z, Cao T and Peng Y (2017). Concurrent CCR7 overexpression and RelB knockdown in immature dendritic cells induces immune tolerance and improves skin-graft survival in a murine model. Cell Physiol Biochem 42:455–468 28578354 10.1159/000477593

[31] Ke N, Su A, Huang W, Szatmary P and Zhang Z (2016). Regulating the expression of CD80/CD86 on dendritic cells to induce immune tolerance after xeno-islet transplantation. Immunobiology 221:803–812 26879762 10.1016/j.imbio.2016.02.002

[32] Ahn Y-H, Hong S-O, Kim JH, Noh KH, Song KH, Lee YH, Jeon JH, Kim DW, Seo JH and Kim TW (2015). The siRNA cocktail targeting interleukin 10 receptor and transforming growth factor-β receptor on dendritic cells potentiates tumour antigen-specific CD8(+) T cell immunity. Clin Exp Immunol 181:164–178 25753156 10.1111/cei.12620 PMC4469167

[33] Wang T, Jiang Q, Chan C, Gorski KS, McCadden E, Kardian D, Pardoll D and Whartenby KA (2009). Inhibition of activation-induced death of dendritic cells and enhancement of vaccine efficacy via blockade of MINOR. Blood 113:2906–2913 19164597 10.1182/blood-2008-08-176354 PMC2662637

[34] Iwata-Kajihara T, Sumimoto H, Kawamura N, Ueda R, Takahashi T, Mizuguchi H, Miyagishi M, Takeda K and Kawakami Y (2011). Enhanced cancer immunotherapy using STAT3-depleted dendritic cells with high Th1-inducing ability and resistance to cancer cell-derived inhibitory factors. J Immunol 187:27–36 21632716 10.4049/jimmunol.1002067

[35] Assi H, Espinosa J, Surprise S, Sofroniew M, Doherty R, Zamler D, Lowenstein PR and Castro MG (2014). Assessing the role of STAT3 in DC differentiation and autologous DC immunotherapy in mouse models of GBM. PLoS One 9:e96318 24806510 10.1371/journal.pone.0096318 PMC4013007

[36] Zhang Q, Hossain DMS, Duttagupta P, Moreira D, Zhao X, Won H, Buettner R, Nechaev S, Majka M, et al. (2016). Serum-resistant CpG-STAT3 decoy for targeting survival and immune checkpoint signaling in acute myeloid leukemia. Blood 127:1687–1700 26796361 10.1182/blood-2015-08-665604 PMC4817311

