# Supplementary material for: MIS416 as a siRNA Delivery System with the Ability to Target Antigen-Presenting Cells
Source: Nucleic Acid Ther. 2018 Aug 1;28(4):225–32. doi: 10.1089/nat.2017.0695 (PMC6080116; doi:10.1089/nat.2017.0695)
Supplement: Supplemental data [file Supp_Data.pdf]

Supplementary Data

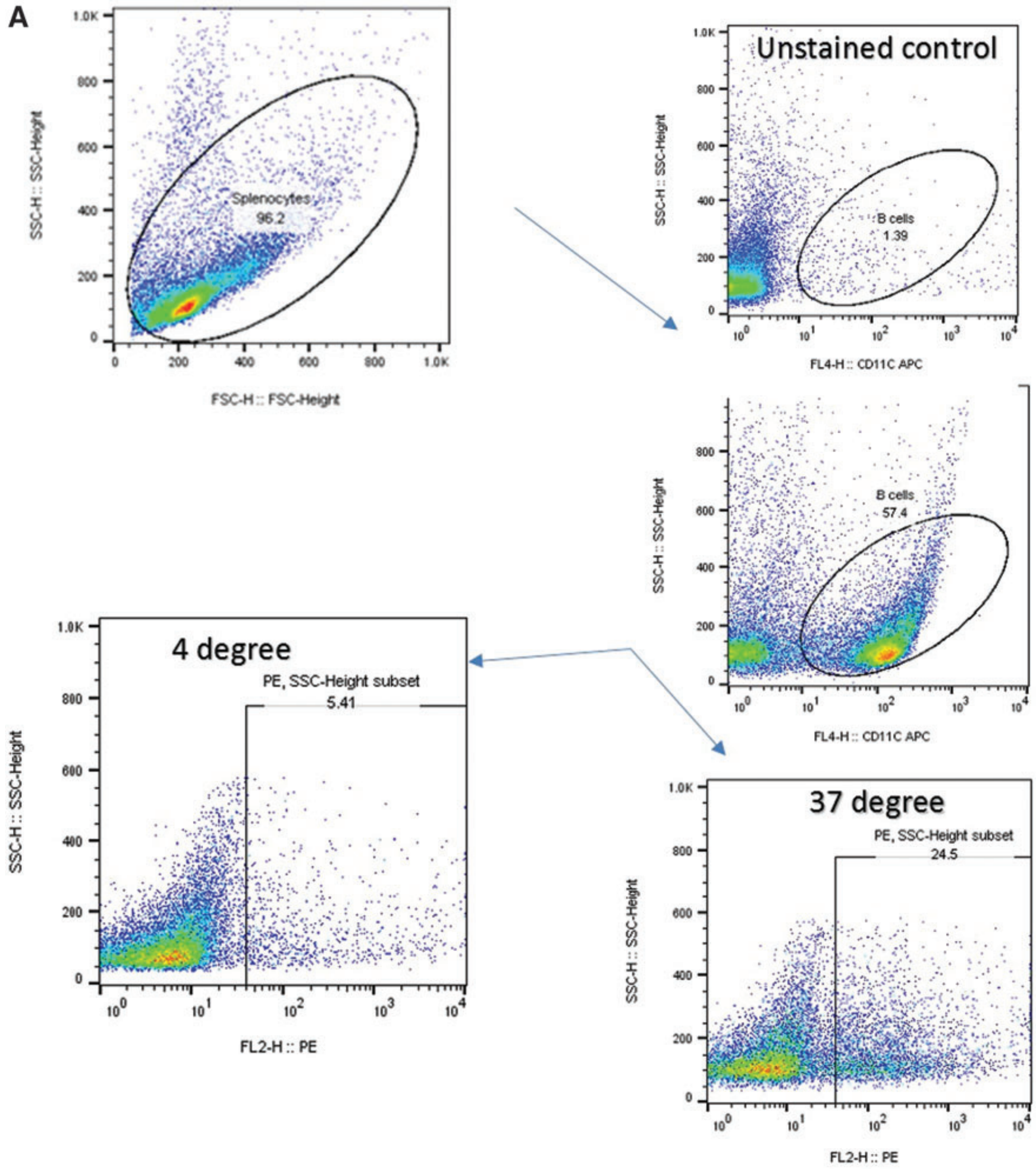

SUPPLEMENTARY FIG. S1. Gating strategy for Figure 1. Splenocytes were gated for dimension and for positivity to the different markers representing different cell populations. **(A)** B cells, **(B)** Macrophages, **(C)** DCs **(D)** Neutrophils, **(E)** T cells. Cells that have internalized MIS416-PE microparticles were identified as PE positive. The bottom left panel represent cells positive for MIS416-PE, where the uptake experiment was conducted at 4°C. The bottom right panel represents a 37°C sample. DC, dendritic cell; PE, phycoerythrin.

(*continued*)

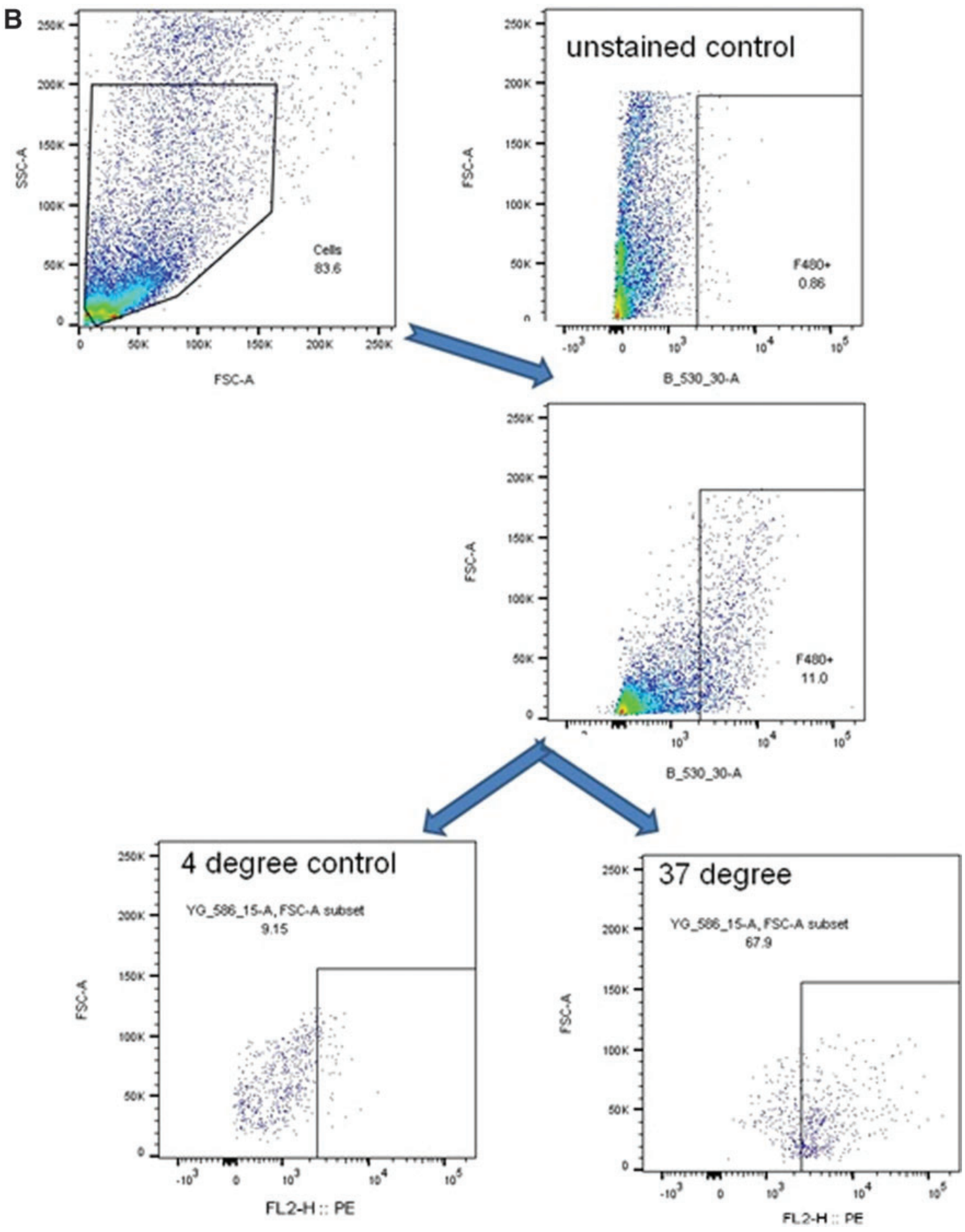

SUPPLEMENTARY FIG. S1. (Continued).

(*continued*)

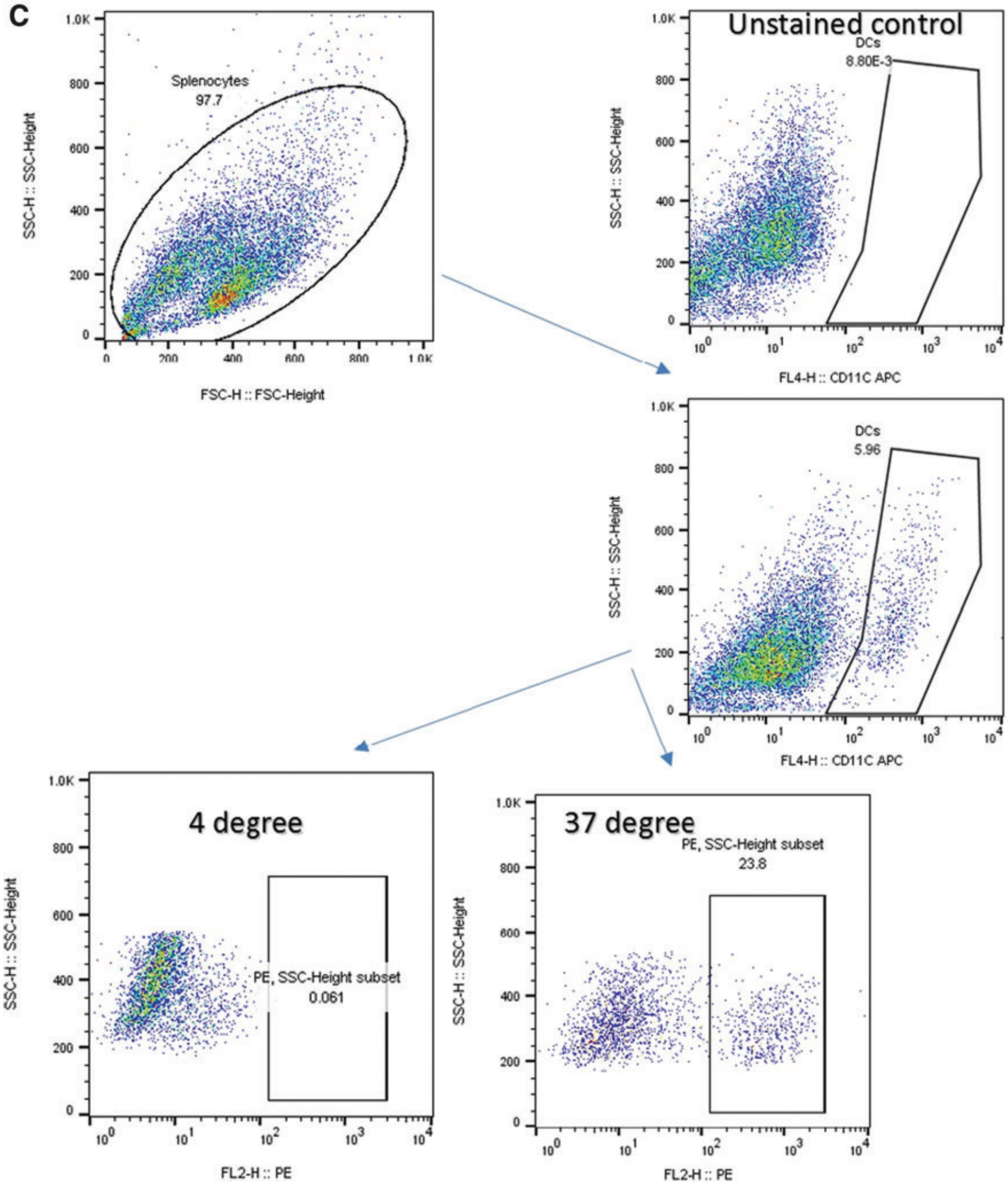

SUPPLEMENTARY FIG. S1. (Continued).

(*continued*)

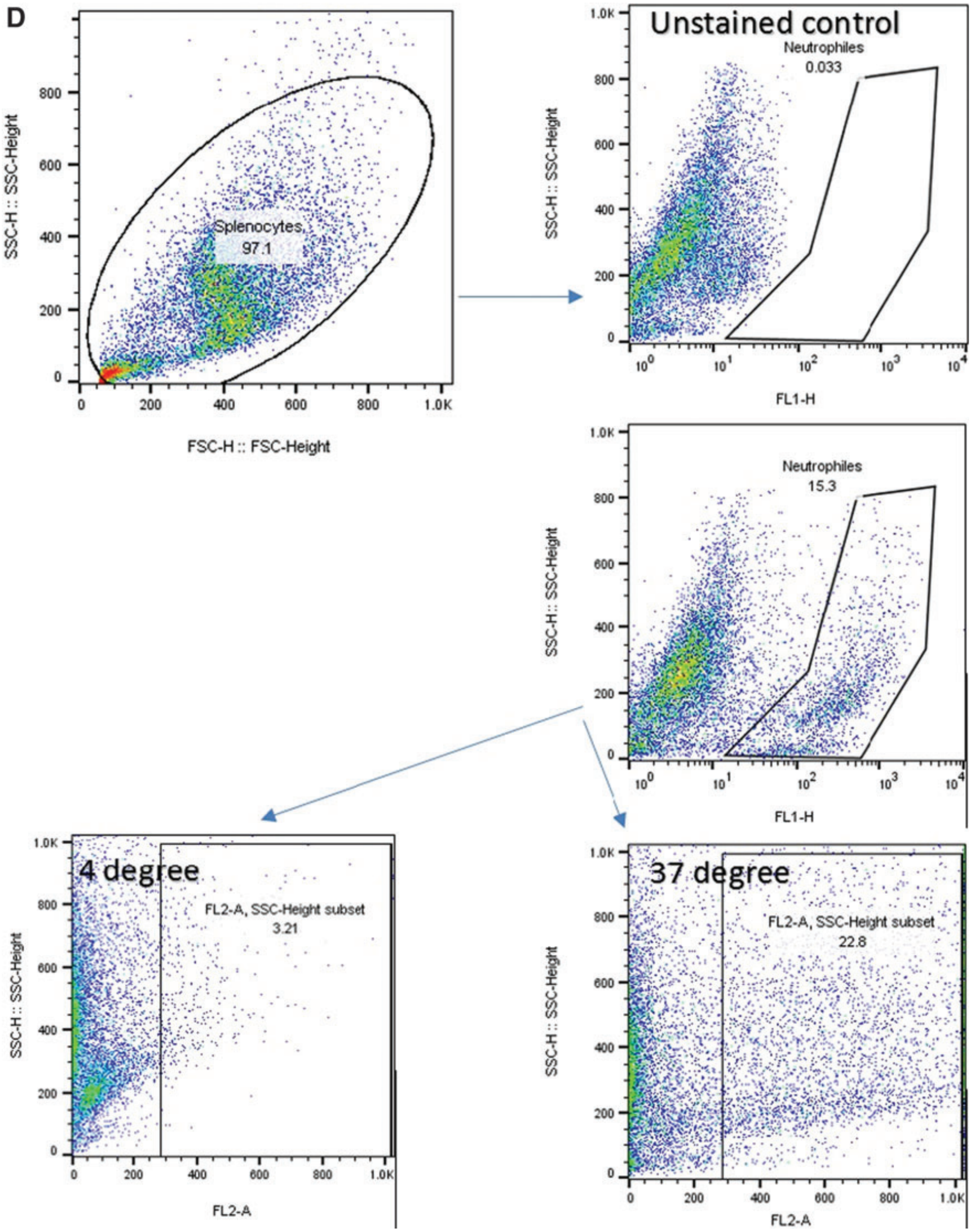

SUPPLEMENTARY FIG. S1. (Continued).

(*continued*)

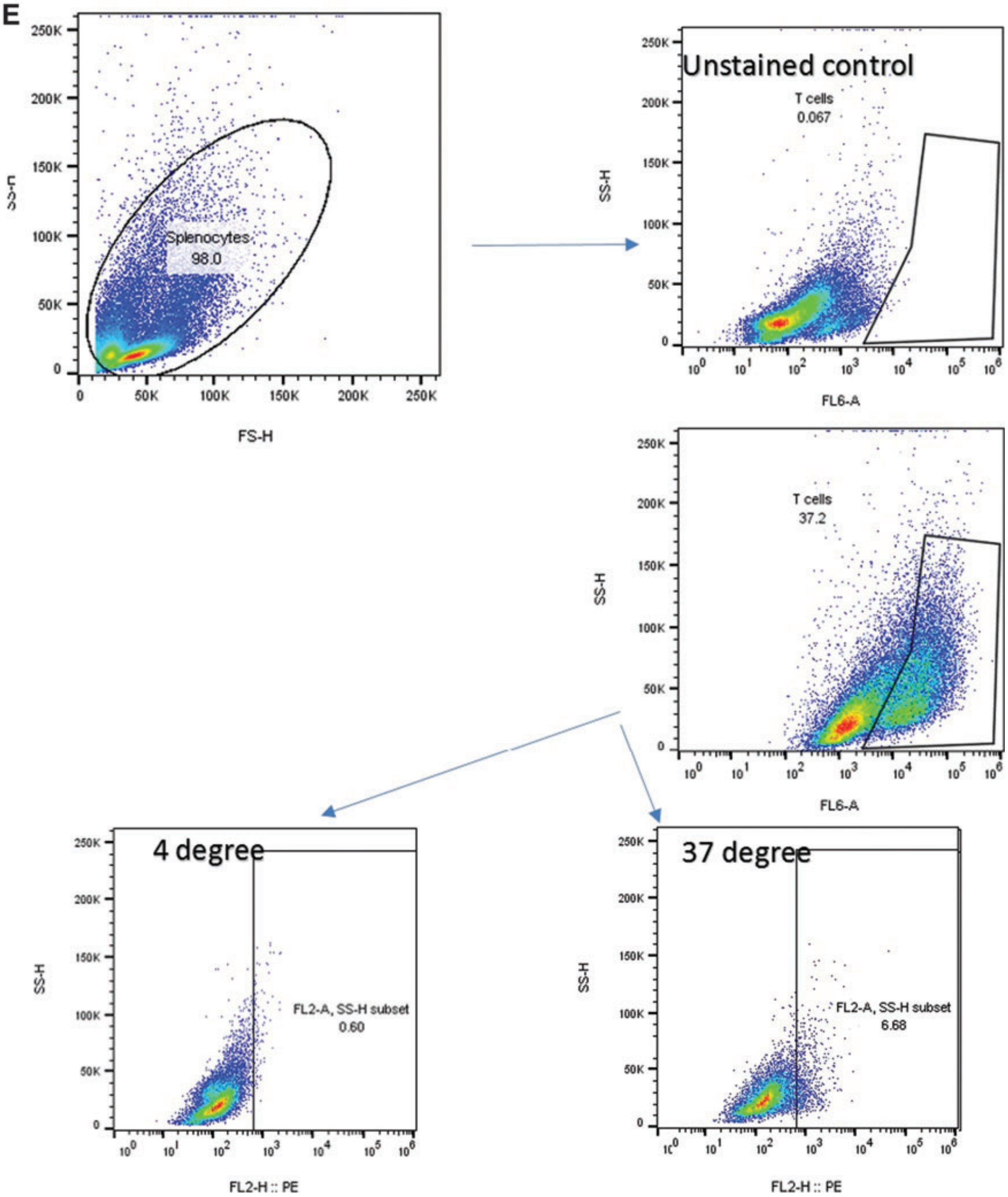

SUPPLEMENTARY FIG. S1. (Continued).

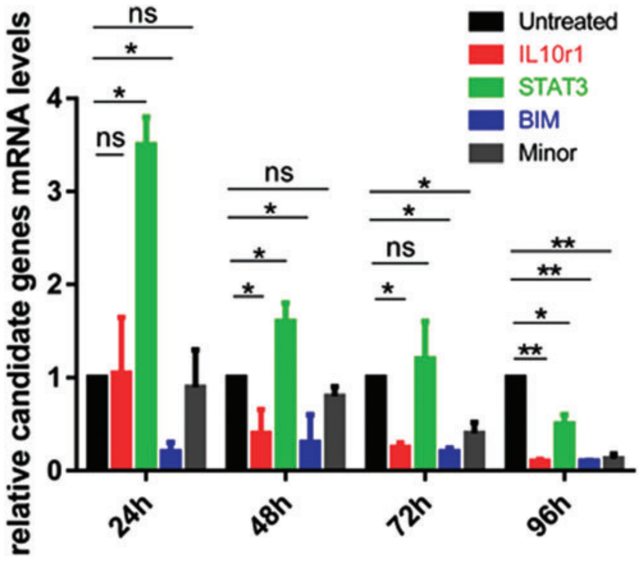

SUPPLEMENTARY FIG. S2. Results from Q-RT-PCR of mRNA levels of *IL10r1, Stat3, Bim, and Minor*. BMDCs were treated with MIS416 for 24, 48, 72, or 96 h and the mRNA levels of the different target genes (*IL10r1, Stat3, Bim,* and *Minor*) were compared with the untreated sample at the same time point. The relative level of expression for all the candidate genes was set to 1 for the untreated sample. Relative quantification was achieved using *Gapdh* as a reference gene. Error bars represent SEM. Results that are not significant are marked with ns, whereas significant results are marked with * depending on the *P* values (*$P < 0.05$, **$P < 0.005$, ***$P < 0.0005$). This experiment was repeated three times. BMDC, bone marrow-derived dendritic cell.

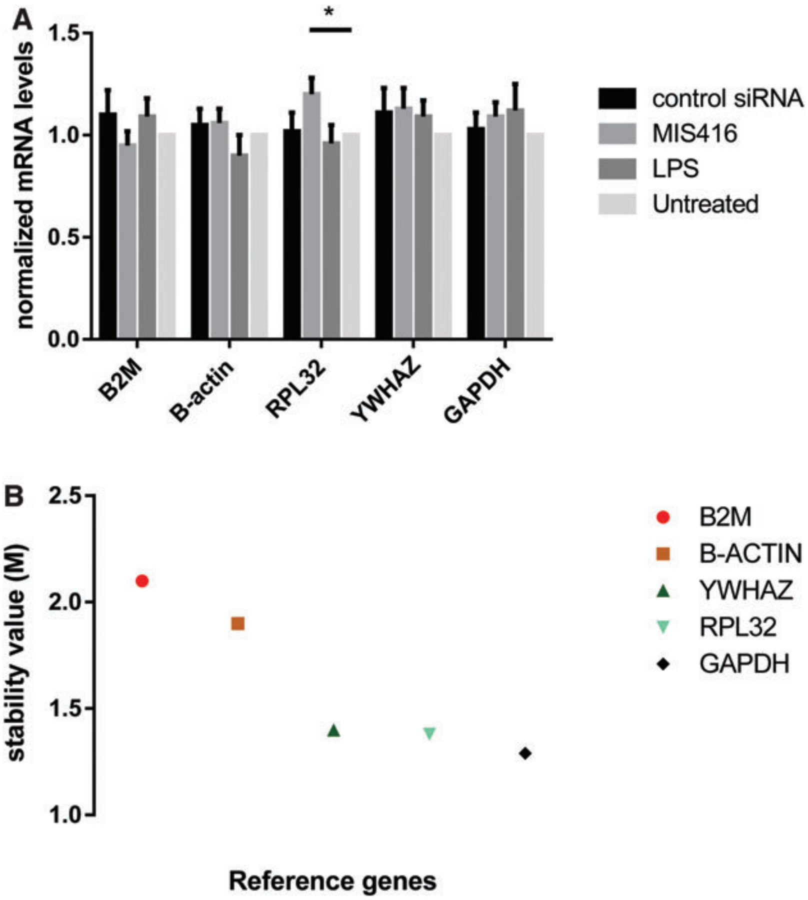

SUPPLEMENTARY FIG. S3. Average expression stability for reference genes. **(A)** DCs were treated with a scrambled siRNA sequence (transfected with RNAiMAX), MIS416, or LPS overnight. Analysis by Q-RT-PCR was performed and results were normalized to the expression of different genes in untreated samples (value set to 1) to compare results. Error bars represent SEM. These experiments were repeated twice. **(B)** Average expression stability values (M) of candidate reference genes by geNorm analysis. DCs were treated with MIS416 and with PBS and RNA was extracted. Analysis by Q-RT-PCR was performed on cDNA generated from total RNA extracted from the cells. The highest M values characterize genes with the least stable expression, indicative of a less optimal reference gene.

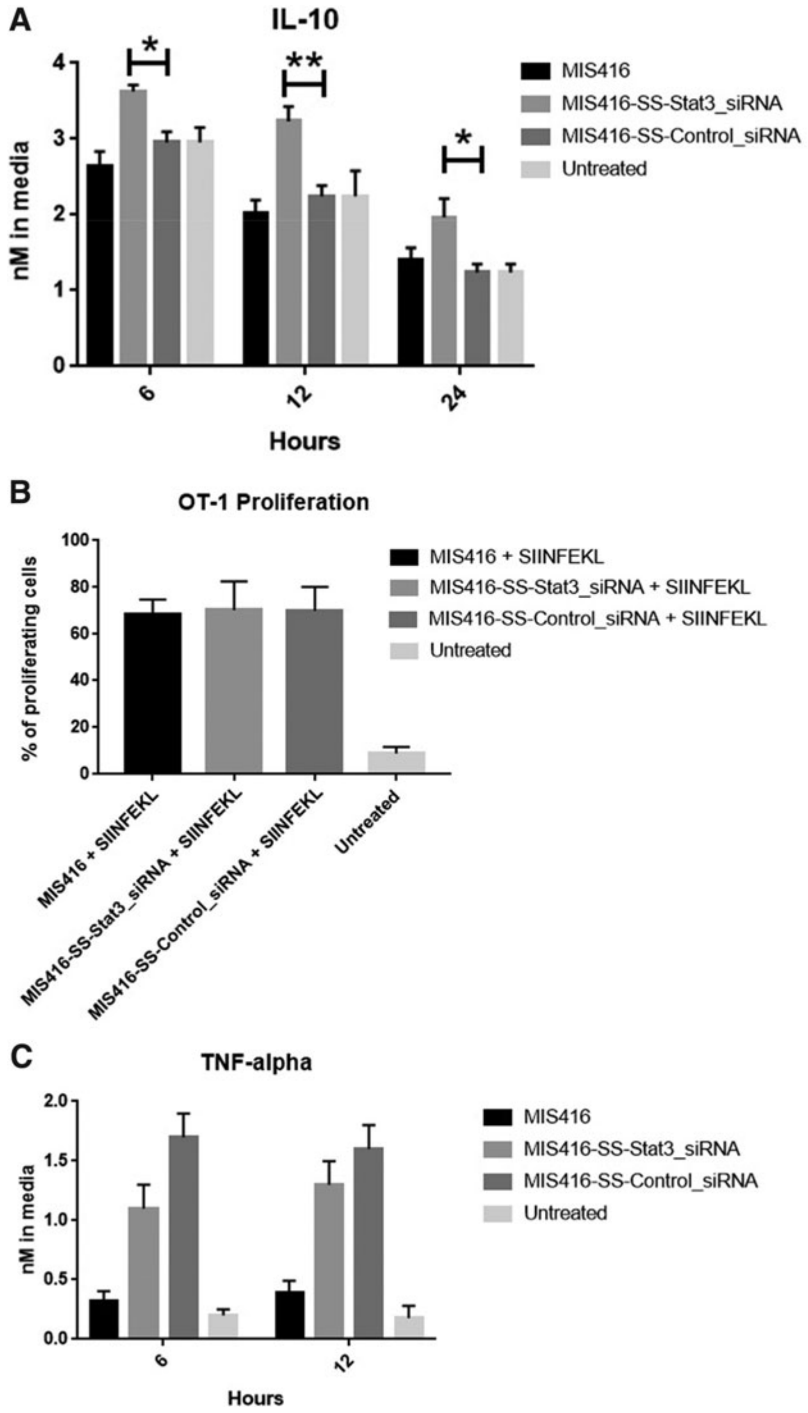

SUPPLEMENTARY FIG. S4. Cytokine expression and OT-1 proliferation in response to treatment with MIS416 or MIS416-SS-siRNA conjugates. **(A)** Graph of ELISA assays of IL-10 cytokine levels in BMDCs at 6, 12, and 24 h following treatment with MIS416, MIS416-SS-Stat3_siRNA, MIS416-SS-control_siRNA, or no treatment. **(B)** Graph of OT-1 T cell proliferation following treatment for 24 h with MIS416 + SIINFEKL, MIS416-SS-Stat3_siRNA + SIINFEKL, MIS416-SS-Control_siRNA + SIINFEKL, or no treatment. **(C)** Graph of TNFα cytokine levels following treatment of BMDCs for 6 or 12 h with MIS416, MIS416-SS-Stat3_siRNA, MIS416-SS-control_siRNA, or no treatment. In all graphs, error bars represent SEM, and results designated with * were significant ($P < 0.05$). Experiments were repeated three times.

Supplementary Table S1. Primers Used in This Study

Primer	*Sequence*	*Reference*
BIM (*BCL2L11*) forward	CGACAGTCTCAGGAGGAACC	[S1]
BIM (*BCL2L11*) reverse	CATTTGCAAACACCCTCCTT	[S1]
STAT3 forward	GGATCGCTGAGGTACAACCC	[S2]
STAT3 reverse	GTCAGGGGTCTCGACTGTCT	[S2]
B2M forward	ATTCACCCCCACTGAGACTG	[S3]
B2M reverse	TGCTATTTCTTTCTGCGTGC	[S3]
β-actin (*ACTB*) forward	CTACAATGAGCTGCGTGTGG	[S4]
β-actin (*ACTB*) reverse	GGTCTCATGGATACCACAGG	[S4]
YWHAZ forward	ACTTGACATTGTGGACATCGGATAC	[S5]
YWHAZ reverse	GTTGGAAGGCCGGTTAATTTTC	[S5]
RPL32 forward	AACGTCAAGGAGCTGGAAGTG	[S6]
RPL32 reverse	GGCTTTGCGGTTCTTGGA	[S6]
Minor forward	AGCAGCTTAAAGGACCACCA	[S7]
Minor reverse	GGGTGTCAAGGAAGAGCTTG	[S7]
IL10r1 *IL10ra* forward	AGGCAGAGGCAGCAGGCC	[S8]
IL10r1 *IL10ra* reverse	TGGAGCCTGGCTAGCTGGTCACAGTAGGTC	[S8]

References

S1. Staton TL, V Lazarevic, DC Jones, AJ Lanser, T Takagi, S Ishii, LH Glimcher. (2011). Dampening of death pathways by schnurri-2 is essential for T-cell development. Nature 472:105–109.

S2. Kim E, M Kim, DH Woo, Y Shin, J Shin, N Chang, YT Oh, H Kim, J Rheey, et al. (2013). Phosphorylation of EZH2 activates STAT3 signaling via STAT3 methylation and promotes tumorigenicity of glioblastoma stem-like cells. Cancer Cell 23:839–852.

S3. Boon K, JK Tomfohr, NW Bailey, S Garantziotis, Z Li, DM Brass, S Maruoka, JW Hollingsworth and DA Schwartz. (2008). Evaluating genome-wide DNA methylation changes in mice by Methylation Specific Digital Karyotyping. BMC Genomics 9:598.

S4. Sun LL, Y Han, JH Chen and YQ Yao. (2008). Down-regulation of HLA-G boosted natural killer cell-mediated cytolysis in JEG-3 cells cultured in vitro. Fertil Steril 90:2398–2405.

S5. Li CG, JE Nyman, AW Braithwaite and MR Eccles. (2011). PAX8 promotes tumor cell growth by transcriptionally regulating E2F1 and stabilizing RB protein. Oncogene 30:4824–4834.

S6. Jeffs AR, AC Glover, LJ Slobbe, L Wang, S He, JA Hazlett, A Awasthi, AG Woolley, ES Marshall, et al. (2009). A gene expression signature of invasive potential in metastatic melanoma cells. PLoS One 4:e8461.

S7. Wang T, Q Jiang, C Chan, KS Gorski, E McCadden, D Kardian, D Pardoll and KA Whartenby. (2009). Inhibition of activation-induced death of dendritic cells and enhancement of vaccine efficacy via blockade of MINOR. Blood 113:2906–2913.

S8. Denning TL, NA Campbell, F Song, RP Garofalo, GR Klimpel, VE Reyes, PB Ernst. (2000). Expression of IL-10 receptors on epithelial cells from the murine small and large intestine. Int Immunol 12:133–139.
